# Editorial: Advancements in biomarker genetics: insights from the genomics era

**DOI:** 10.3389/fmolb.2026.2014518

**Published:** 2026-10-16

**Authors:** Simone L. Cree, Moritz Lassé, Anna P. Pilbrow

**Affiliations:** 1 Christchurch Heart Institute, Department of Medicine, University of Otago, Christchurch, New Zealand; 2 Department of Medicine, and Hamburg Center for Kidney Health (HCKH), University Medical Center Hamburg-Eppendorf, Hamburg, Germany

**Keywords:** biomarkers, equity, genomics, population diversity, reference ranges

The genomic era is changing not only how biomarkers are discovered, but also how they are interpreted. Clinical biomarkers have traditionally been evaluated against population-derived reference intervals and diagnostic thresholds. Yet an expanding body of evidence demonstrates that genetic variation, epigenetic regulation, environmental exposures, and their interactions can influence biomarker concentrations, disease susceptibility, and therapeutic response. At the same time, advances in sequencing technologies are allowing these layers of biological variation to be examined with unprecedented resolution. The current challenge is therefore shifting from applying biomarkers using a ‘one size fits all’ approach to understanding the biological context that determines what a biomarker means for an individual.

The Research Topic “*Advancements in Biomarker Genetics: Insights from the Genomics Era*” brings together eight contributions illustrating this transition. Collectively, these studies span genetic and epigenetic biomarkers, long-read sequencing, pharmacogenomics, metabolic biomarkers, circulating bacterial DNA, and population-specific genetic associations. Despite their diverse clinical settings, they converge on a common principle: biomarker interpretation increasingly requires consideration of genomic diversity within and between populations.

This is particularly evident in the contribution by van der Laan et al., “*Nanopore sequencing enables combined detection of USP7 variants and a known Hao-Fountain syndrome episignature*”. Using Oxford Nanopore long-read sequencing in 5 individuals with Hao-Fountain syndrome, the authors identified pathogenic *USP7* single-nucleotide variants, structural deletions and a characteristic DNA methylation episignature. The study demonstrates the potential for a single sequencing platform to integrate genomic and epigenomic information that has traditionally required separate assays. Such approaches point towards a future in which several different types of markers can be measured a single assay, potentially leading to better risk estimates and reduced cost.

The epigenetic dimension of biomarker discovery is developed further by Munns et al., “*Type-2 diabetes epigenetic biomarkers: present status and future directions for global and Indigenous health*”. Their review highlights the potential of DNA methylation signatures for risk prediction and monitoring in type 2 diabetes, while identifying important limitations in reproducibility, genome coverage and population representation. Importantly, they argue for sequencing-based methylation profiling, larger longitudinal cohorts, and the meaningful inclusion of genetically diverse and Indigenous populations. This contribution emphasizes that technological innovation and health equity must progress together if precision medicine is to deliver broadly applicable benefits.

The importance of population context is also evident in Saed et al., “*Association between CTLA-4 gene polymorphisms and Type 1 diabetes in Kurdish patients*”. In this preliminary study, the G allele of the *CTLA-4* +49 A>G (rs231775) variant was associated with increased susceptibility to type 1 diabetes in a Kurdish population in Iraq (52 patients, 21 healthy controls), whereas the −318 C>T variant was not. These polymorphisms may influence susceptibility to type 1 diabetes by altering expression and cell-surface availability of CTLA-4, an immune checkpoint receptor that acts as a brake on T-cell activation, thereby potentially permitting enhanced autoreactive T-cell-mediated destruction of pancreatic β-cells. Although this finding requires confirmation in larger cohorts, the study illustrates the value of extending genetic association research to populations historically underrepresented in genomic studies.

Population-specific evaluation is equally relevant to conventional biochemical biomarkers. In their study “*Associations between glycated albumin and current measures of glycaemic control in Saudi adults*”, Bahijri et al. found that glycated albumin correlated with established measures of glycaemic status and distinguished normoglycaemia from intermediate hyperglycaemia and diabetes in 132 adults from Saudi Arabia. Their findings support further evaluation of glycated albumin as an alternative or complementary glycaemic biomarker while emphasizing the need to establish appropriate ranges in the population in which it will be used.

The relationship between genotype and clinical phenotype is explored from another perspective by Ji et al., “*Correlation of UGT1A1 genotypes with newborn hyperbilirubinemia using newborn genetic screening*”. Among 115 newborns undergoing genetic screening, biallelic or multi-allelic *UGT1A1* variants were associated with mildly higher transcutaneous bilirubin concentrations at 6 weeks, suggesting slower postnatal bilirubin clearance. However, clinically significant neonatal hyperbilirubinemia was uncommon and did not differ significantly between variant carriers and controls. The study therefore illustrates that the link between detectable genotype–phenotype associations and prediction of clinically significant variants is not always straight forward in genomic biomarker research.

The need for similar caution is reinforced by Wang et al. in their study “*Association between the TNF-α-308G/A and TNF-α-238G/A polymorphisms and systemic lupus erythematosus susceptibility: an updated meta-analysis*”. Although conventional meta-analysis of 53 studies identified several statistically significant associations, evaluation using prediction intervals and Bayesian false-discovery probability indicated that these were less-credible positive findings. This work provides a timely reminder that statistical association alone is insufficient for biomarker discovery; reproducibility, effect size, heterogeneity, population structure, and the probability of false-positive findings must all be considered.

Genomic information may also help personalize treatment. In “*Gene polymorphisms associated with immunosuppressant adverse effects in systemic lupus erythematosus: a narrative review*”, Hamdani et al., summarize variants associated with adverse effects from methotrexate, azathioprine, cyclophosphamide, and mycophenolate mofetil across 23 studies. Their review illustrates the potential of pharmacogenomic biomarkers to move precision medicine beyond disease susceptibility towards drug selection, dosage optimization, and avoidance of treatment toxicity.

Finally, in their review “*Circulating bacterial DNA in cardiovascular disease*”, Appleby and Purcell broaden the genomic biomarker concept beyond the human genome. They highlight circulating bacterial DNA as a potential diagnostic and prognostic marker in cardiovascular disease and examine microbial translocation as one possible biological source. However, heterogeneity in study populations, sample types, and analytical methodologies currently limits clinical translation, emphasizing the need for larger cohorts and methodological standardization.

Together, these contributions reveal several priorities for the next phase of biomarker discovery. The field is moving beyond single genetic variants and isolated measurements towards integrated, multi-modal markers including genomic, epigenomic, proteomic, microbial, environmental, imaging and clinical information. Associations require rigorous replication and demonstration of clinical utility. Reference intervals and diagnostic thresholds should be evaluated across diverse populations rather than assumed to be universally transferable. Equally importantly, the populations most affected by disease must be appropriately represented in biomarker discovery studies, with genomic research conducted according to principles of meaningful engagement, equity, and appropriate data sovereignty and governance.

The genomics era therefore offers more than an expanding catalogue of biomarkers. It provides an opportunity to understand why biomarkers differ among individuals and populations, and to incorporate that knowledge into more precise clinical interpretation. The studies in this Research Topic illustrate both the promise and the challenges involved in that transition. Ultimately, the goal is not simply to measure biomarkers more accurately, but to interpret them more meaningfully—linking genomic diversity to diagnosis, prognosis, therapeutic response, and equitable precision medicine.

